# Preoxygenation When Standard Approaches Fail: Phenotype-Based Strategies for High-Risk Emergent Intubations

**DOI:** 10.3390/jcm15072477

**Published:** 2026-03-24

**Authors:** Laura Gutierrez, Abhinandan Chittal, Sydney Fiore, Perry Tiberio

**Affiliations:** 1 Division of Pulmonary and Critical Care, Allegheny Health Network, Pittsburgh, PA 15212, USA; laura.gutierrez@ahn.org (L.G.); abhinandan.chittal@ahn.org (A.C.); 2 College of Medicine, Drexel University, Philadelphia, PA 19104, USA; sac523@drexel.edu

**Keywords:** pre-oxygenation, intubation, airway management

## Abstract

Emergent tracheal intubation in critically ill patients is a common, yet high-risk, intervention. It is frequently complicated by peri-intubation hypoxemia, hemodynamic instability, and metabolic derangements that increase the risk of arrhythmias, hypotension, cardiac arrest, and death. Because the highest-risk interval often occurs in the minutes surrounding induction, when apnea, derecruitment, and abrupt cardiopulmonary shifts converge, oxygenation failure frequently reflects a mismatch between preoxygenation strategy and the underlying physiology rather than inadequate oxygen delivery alone. This review proposes a phenotype-based approach to peri-intubation oxygenation and focuses on four high-risk phenotypes in whom standard preoxygenation strategies commonly fail: obesity, neuromuscular disease, right ventricular dysfunction or pulmonary hypertension, and post-operative respiratory failure with altered respiratory mechanics or airway anatomy. We summarize the key mechanisms that shorten safe apnea time, including reduced functional residual capacity, intrapulmonary shunt, elevated oxygen consumption, rapid derecruitment after induction, and impaired oxygenation–hemodynamics coupling. We then compare preoxygenation modalities as physiologic tools, including facemask oxygen, high-flow nasal cannula (HFNC), noninvasive ventilation (NIV), and controlled bag-mask ventilation (BMV), and integrate contemporary randomized trial evidence that informs bedside selection and combination of these approaches. Finally, we synthesize these concepts into a practical, physiology-informed framework to guide clinicians in choosing and troubleshooting preoxygenation strategies in high-risk patients undergoing emergent intubation.

## 1. Introduction

Emergent intubation in critically ill patients remains a high-risk intervention, where peri-intubation hypoxemia and cardiovascular instability serve as primary drivers of morbidity and mortality. Unlike elective airway management in the operating room, where patients are typically fasted and hemodynamically optimized, ICU and emergency department intubations frequently occur in patients with severe hypoxemia, limited cardiopulmonary reserve, and metabolic derangements. These urgent conditions restrict the time available for optimization and amplify the physiologic risks associated with apnea. Peri-intubation hypoxemia occurs in approximately 9% to 15% of critically ill intubations, with cardiac arrest occurring in 2% to 3%, illustrating the narrow margin for error in this population [1]. Given that cardiac arrest during airway management is associated with profound morbidity, its absolute avoidance is a paramount clinical priority. This is especially critical, as recent evidence suggests that resuscitative efforts, including repeated doses of epinephrine, are associated with poor neurologic outcomes and diminished overall survival following arrest [2].

The highest-risk interval is not laryngoscopy itself but the peri-induction window, the minutes immediately before and after induction, when apnea, rapid alveolar derecruitment, and abrupt hemodynamic shifts converge. During this interval, critically ill patients may progress from acceptable oxygen saturations to severe hypoxemia or cardiovascular collapse within seconds. This vulnerability often reflects a mismatch between the chosen preoxygenation strategy and the dominant physiologic constraint limiting safe apnea time, rather than a failure of oxygen delivery alone [3].

Preoxygenation is therefore not a uniform preparatory step, but a physiology-dependent intervention whose success relies on addressing the specific mechanisms that shorten safe apnea time in each patient. While recent randomized trials have clarified the relative performance of noninvasive ventilation (NIV), high-flow nasal cannula (HFNC), and bag-mask ventilation, device-centric recommendations do not fully capture why preoxygenation succeeds in some patients and fails catastrophically in others [4].

In this review, we reframe peri-intubation oxygenation through a phenotype-based lens, emphasizing the peri-induction window as a modifiable determinant of outcome. Recent meta-analyses [4] have compared pre-oxygenation devices across critically ill populations but are largely centered around specific device performance rather than underlying physiology, which may influence the success or failure of intubation in individual patient populations. A phenotype-based approach is necessary because a patient’s comorbidities and baseline physiology fundamentally dictate their response to induction, representing a significant departure from the pre-optimized conditions typical of elective operating room intubations. We first summarize the core physiologic mechanisms that accelerate desaturation and collapse during emergent intubation. We then outline how preoxygenation strategies function as physiologic tools that target specific failure mechanisms. Finally, we apply this framework to four high-risk phenotypes: obesity, neuromuscular disease, right ventricular failure or pulmonary hypertension, and post-operative respiratory failure. These four specific phenotypes have been selected as they represent common clinical scenarios that have distinct physiologic constraints on safe apnea time. Each constraint can lead to reduced lung volume, impaired ventilatory reserves, shunt physiology, and hemodynamic effects on oxygenation. By translating complex physiology into bedside strategies, clinicians can anticipate failure modes rather than react to them.

## 2. Methods

We conducted an evidence-based search using PubMed, the Cochrane Library, and Google Scholar to compile this narrative review. Data were synthesized from multiple domains, including the intensive care, anesthesia, cardiology, and emergency medicine literature. The primary objective of this review is to propose a phenotype-based framework for peri-intubation management in the clinical setting. To achieve this, we performed a targeted search using terms such as “preoxygenation,” “intubation,” “peri-intubation,” and “airway management”. These were combined with specific keywords including “high-flow nasal cannula,” “noninvasive ventilation,” “obesity,” “neuromuscular disease,” “right ventricular dysfunction,” and “post-operative respiratory failure”. We analyzed findings based on their clinical relevance and physiologic insight as applied to critically ill adult patients. In keeping with the narrative nature of this review, we prioritized the synthesis of mechanism-to-strategy matching over a systematic cataloging of all the available literature. As this manuscript is a narrative synthesis, and not a systematic review or meta-analysis, no formal statistical analysis was performed.

## 3. Core Physiologic Mechanisms That Shorten Safe Apnea Time

Severe peri-intubation hypoxemia in critically ill patients arises from a predictable interaction of the respiratory, metabolic, and hemodynamic factors that dramatically shorten safe apnea time [1]. While these mechanisms frequently coexist, their relative contribution varies by clinical phenotype, creating distinct patterns of preoxygenation failure that can be anticipated and targeted. These are summarized in Table 1.

### 3.1. Reduced Functional Residual Capacity and Rapid Derecruitment

Functional residual capacity (FRC) represents the principal intrapulmonary oxygen reservoir during apnea. In critical illness, FRC is commonly reduced by atelectasis, pulmonary edema, supine positioning, obesity, neuromuscular disease, and postoperative respiratory mechanics. Loss of diaphragmatic tone following induction further reduces transpulmonary pressure, promoting the rapid collapse of dependent lung units. Even with high inspired oxygen fractions, a diminished and unstable alveolar reservoir cannot sustain arterial oxygenation during apnea, leading to precipitous desaturation [5,6,7].

### 3.2. Intrapulmonary Shunt and Ventilation–Perfusion (V/Q) Mismatch

Conditions that precipitate emergent intubation, such as pneumonia, ARDS, aspiration, and pulmonary edema, often produce substantial intrapulmonary shunt. In these states, supplemental oxygen enriches ventilated alveoli but cannot correct gas exchange across collapsed or flooded lung units. Induction-associated derecruitment further increases the shunt fraction, rendering oxygen enrichment alone insufficient to meaningfully prolong safe apnea time [3,6].

### 3.3. Elevated Oxygen Consumption and Limited Metabolic Reserve

Critical illness frequently increases whole-body oxygen consumption due to fever, sepsis, agitation, acidosis, and heightened work of breathing. Sedation and neuromuscular blockade eliminate respiratory effort but do not reduce systemic oxygen demand. As a result, oxygen stores are depleted rapidly during apnea, particularly in patients with shock, severe metabolic acidosis, or agitation, where tissue oxygen extraction is already maximal [8,9].

### 3.4. Oxygenation–Hemodynamic Coupling and Limited Cardiopulmonary Reserve

Hemodynamic instability profoundly constrains patients’ tolerance of apnea. Reduced cardiac output and mixed venous oxygen saturation limit the buffering capacity of the pulmonary circulation, while induction agents and positive-pressure ventilation may further reduce preload and systemic arterial pressure. In patients with pulmonary hypertension or right ventricular (RV) failure, even brief hypoxemia or hypercapnia can acutely increase pulmonary vascular resistance, precipitating RV ischemia, interventricular septal shift, and abrupt cardiovascular collapse, often before severe oxygen desaturation is observed [10,11].

### 3.5. Procedural Amplifiers of Physiologic Collapse

Sedation, neuromuscular blockade, prolonged laryngoscopy attempts, suboptimal positioning, and interruptions in oxygen delivery compound underlying physiologic vulnerability. These factors accelerate derecruitment and oxygen depletion during a window in which critically ill patients may tolerate only seconds of apnea [5,6,7].

Together, these mechanisms define the physiologic limits of safe apnea. Preoxygenation strategies succeed only when they address the dominant constraint present in each patient.

## 4. Mechanism-to-Strategy Matching in the Peri-Induction Window

As outlined above, effective preoxygenation in critically ill patients requires more than maximizing the inspired oxygen concentration. Instead, it requires intentional matching of strategy to mechanism, with the goal of expanding oxygen reserves, stabilizing lung volume, maintaining oxygen delivery during apnea, and minimizing hemodynamic perturbation during induction [12].

Broadly, preoxygenation strategies function through four complementary physiologic targets:

1. Expansion of alveolar oxygen stores. Oxygen enrichment replaces alveolar nitrogen, increasing the oxygen content of the existing FRC. This target is necessary but rarely sufficient in patients with reduced lung volume or shunt physiology [12];
2. Stabilization and recruitment of lung volume. Positive airway pressure delivered via NIV or CPAP increases FRC, recruits atelectatic lung units, and reduces shunt fraction. This mechanism is necessary in patients with low baseline lung volume or rapid derecruitment after induction [12];
3. Maintenance of oxygenation during apnea. Continuous oxygen delivery via HFNC or nasal cannula during laryngoscopy supports apneic oxygenation by preserving the alveolar–arterial diffusion gradient. Its benefit is greatest when lung compliance is preserved and the shunt fraction is modest [13];
4. Prevention of derecruitment during induction. Gentle bag-mask ventilation or continuation of NIV during the induction–laryngoscopy interval preserves end-expiratory lung volume and slows oxygen depletion in patients with minimal reserve [14].

No single modality addresses all four targets. Consequently, multimodal strategies such as NIV for recruitment followed by HFNC for apneic oxygenation, or recruitment-focused preoxygenation combined with controlled ventilation during induction are often required in high-risk patients.

Equally important, preoxygenation strategy selection must account for oxygenation–hemodynamic coupling. In patients with shock or RV failure, excessive intrathoracic pressure, prolonged apnea, hypercapnia, or induction-associated hypotension may be more dangerous than hypoxemia alone. In these settings, minimizing apnea duration and preserving preload and systemic arterial pressure are integral components of successful preoxygenation [10].

This mechanism-based framework provides the foundation for a phenotype-directed approach. In the sections that follow, we apply these principles to four high-risk clinical phenotypes in whom standard preoxygenation frequently fails, integrating the physiologic rationale with the available evidence to guide bedside strategy selection and troubleshooting.

## 5. Preoxygenation Modalities as Physiologic Tools

Preoxygenation modalities are often discussed as interchangeable techniques distinguished primarily by oxygen flow rates or interface type. In critically ill patients, however, their effectiveness is determined by which physiologic constraints they address and which they do not. Rather than cataloging devices, this section frames commonly used modalities according to their dominant physiologic effects, limitations, and failure modes during the peri-induction window. Key randomized trials informing these strategies are summarized in Table 2.

### 5.1. Facemask Oxygen and Conventional Preoxygenation

Facemask oxygen delivered during spontaneous breathing remains the most widely available method of preoxygenation. When applied with an adequate seal and high oxygen flow, it can achieve substantial alveolar denitrogenation and expand oxygen content within the existing functional residual capacity (FRC) [20].

However, conventional facemask preoxygenation does not recruit collapsed lung units or stabilize end-expiratory lung volume. Its effectiveness is therefore highly dependent on preserved lung compliance, minimal shunt physiology, and adequate baseline FRC. Mask leak, high inspiratory demand, agitation, and supine positioning further limit its reliability in critically ill patients. As a result, facemask oxygen alone is often insufficient in patients with obesity, neuromuscular disease, acute hypoxemic respiratory failure, or postoperative atelectasis, in whom rapid derecruitment rather than oxygen enrichment is the dominant cause of desaturation [21].

Physiologic role: Oxygen enrichment only [21].
Primary limitation: No recruitment; highly sensitive to leak and derecruitment [21].

### 5.2. Noninvasive Ventilation and Positive Airway Pressure Strategies

NIV provides the most comprehensive, single-modality, physiologic preoxygenation by combining a high oxygen concentration with positive airway pressure. By recruiting atelectatic lung units, restoring FRC, and reducing intrapulmonary shunt, NIV directly addresses the dominant mechanisms of preoxygenation failure in many critically ill patients [15].

Randomized trials in ICU populations consistently demonstrate that NIV reduces the incidence and depth of peri-intubation hypoxemia compared with conventional oxygen strategies, particularly in patients with moderate-to-severe hypoxemia or reduced lung compliance. Its benefit derives from lung volume stabilization rather than oxygen enrichment alone [19].

Limitations include mask intolerance, contraindications related to aspiration risk or facial pathology, and potential hemodynamic effects from increased intrathoracic pressure. In patients with shock, pulmonary hypertension, or right ventricular dysfunction, NIV must be applied cautiously, using the lowest effective pressures and paired with proactive hemodynamic support [22,23].

Physiologic role: Recruitment, shunt reduction, FRC restoration [15].
Primary limitation: Tolerance, aspiration risk, hemodynamic effects [22,23].

### 5.3. High-Flow Nasal Cannula

HFNC delivers heated, humidified oxygen at flow rates sufficient to minimize entrainment of ambient air and maintain a high inspired oxygen concentration during spontaneous breathing. Its distinguishing advantage is the ability to remain in place throughout laryngoscopy, enabling continuous oxygen delivery and supporting apneic oxygenation during the induction–laryngoscopy interval [24].

HFNC provides minimal and inconsistent positive airway pressure and does not reliably recruit atelectatic lung units. Consequently, while HFNC may improve oxygenation and prolong safe apnea time in patients with preserved lung compliance or mild hypoxemia, it is often insufficient as a standalone strategy in patients with severe shunt physiology, obesity-related atelectasis, or markedly reduced physiologic reserve [25,26].

HFNC is therefore best understood as a continuity tool—maintaining oxygen delivery when mask-based interfaces must be removed—rather than a recruitment strategy [24].

Physiologic role: Continuous oxygen delivery; apneic oxygenation [24].
Primary limitation: Limited recruitment; reduced efficacy in severe shunt [26].

### 5.4. Bag-Mask Ventilation During Induction

BMV during the interval between induction and laryngoscopy addresses a critical and often overlooked failure point: early oxygen depletion and derecruitment immediately after loss of spontaneous ventilation. Controlled, gentle BMV replenishes the oxygen stores consumed during early apnea and preserves alveolar patency during the induction–laryngoscopy interval [14].

Recent randomized trials demonstrate that carefully delivered BMV did not increase aspiration events and was associated with higher nadir oxygen saturation during intubation. These findings challenge the routine avoidance of ventilation during rapid sequence induction in critically ill patients with limited reserve [14].

BMV is particularly valuable in patients who tolerate apnea poorly, such as those with obesity, neuromuscular disease, or severe hypoxemia, when even brief oxygen interruption may precipitate rapid desaturation. Effective use requires attention to airway patency, often facilitated by oral airway placement, and avoidance of excessive tidal volumes or inspiratory pressures [27].

Physiologic role: Prevents early derecruitment; replenishes oxygen during induction [14].
Primary limitation: Requires skill; airway patency and is seal dependent [14,27].

### 5.5. Positioning as a Foundational Adjunct

Patient positioning is a universal, low-risk intervention that amplifies the effectiveness of all preoxygenation modalities. Head-elevated or ramped positioning increases FRC, reduces dependent lung compression, improves upper airway patency, and facilitates mask seal and laryngoscopy [28].

Positioning is particularly important in patients with obesity, neuromuscular disease, or postoperative respiratory compromise, in whom supine positioning accelerates atelectasis and shortens safe apnea time. Although positioning alone does not increase the alveolar oxygen content, its impact on respiratory mechanics makes it an essential component of preoxygenation rather than a supplemental maneuver [29].

Physiologic role: Improves lung mechanics and airway patency [28].
Primary limitation: None when feasible; requires planning and equipment [28,29].

### 5.6. Combining Modalities to Address Multiple Failure Mechanisms

In critically ill patients, preoxygenation failure rarely reflects a single physiologic deficit. Combining complementary strategies, such as NIV for recruitment followed by HFNC for apneic oxygenation, or recruitment-focused preoxygenation paired with controlled bag-mask ventilation during induction, allows clinicians to address multiple mechanisms simultaneously. These multimodal approaches are particularly valuable in patients with severe hypoxemia, obesity, or neuromuscular disease, where a single modality may be insufficient [16]. The challenge is not the availability of techniques, but the intentional selection and sequencing based on the dominant physiologic constraints present in each patient.

With an understanding of how preoxygenation modalities function as physiologic tools, the next step is determining which combination best matches a given patient’s failure profile. The following section applies this framework to four high-risk phenotypes in whom standard approaches frequently fail, integrating the physiologic rationale with the available clinical evidence to guide strategy selection and troubleshooting.

## 6. Phenotype-Based Application

**Figure 1:**
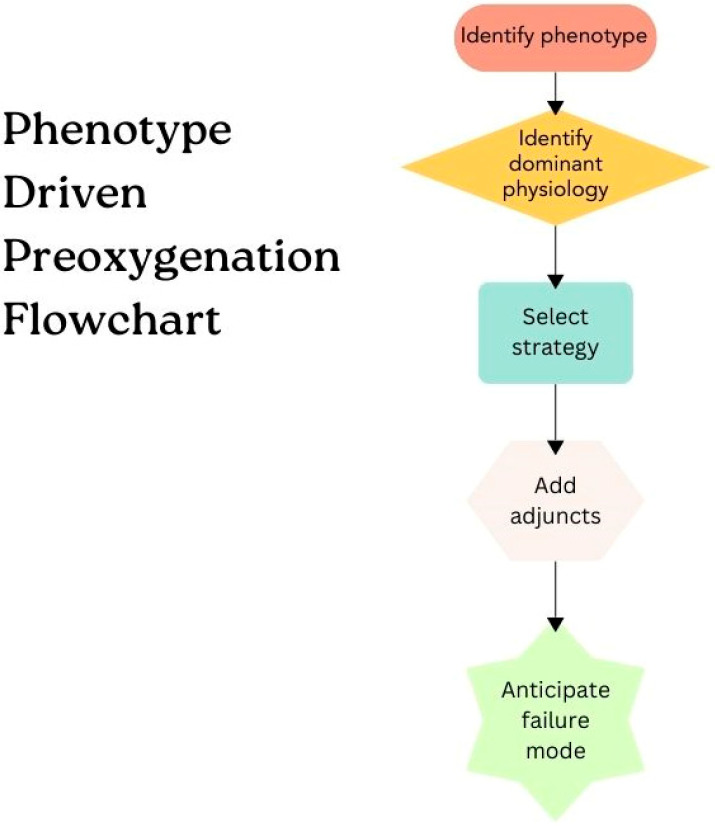
Phenotype-driven preoxygenation flowchart. The flowchart is an original conceptual framework created by the authors.

Together, these physiologic principles highlight that preoxygenation success depends less on the device itself than on whether the chosen strategy addresses the dominant constraint limiting safe apnea time. In high-risk patients, failure most often reflects a mismatch between approach and underlying physiology, rather than inadequate oxygen delivery alone. In the following sections, we apply this framework to four clinical phenotypes in whom standard strategies commonly fail, integrating the physiologic rationale with the available evidence to guide strategy selection, sequencing, and troubleshooting during emergent intubation. These are summarized in Table 3, and an overall workflow has been proposed in Figure 1.

### 6.1. Obesity

#### 6.1.1. Why Preoxygenation Fails

Obesity markedly shortens safe apnea time during emergent intubation due to chronically reduced functional residual capacity and rapid alveolar derecruitment following induction. Increased abdominal mass and reduced chest wall compliance promote early closure of dependent lung units, particularly in the supine position, while loss of diaphragmatic tone after sedation accelerates atelectasis. Elevated metabolic demand further increases oxygen consumption during apnea. As a result, oxygen enrichment alone frequently fails to prevent rapid desaturation, despite apparently adequate preoxygenation [30].

#### 6.1.2. Dominant Physiologic Constraints

- Reduced FRC with early airway closure;
- Rapid derecruitment following induction;
- Increased oxygen consumption;
- Dependent-lung shunt physiology.

Together, these constraints favor strategies that actively recruit and stabilize lung volume rather than oxygen enrichment alone [31].

#### 6.1.3. Evidence and Applicability

Direct evidence specific to emergent intubation in obese critically ill adults is limited. However, data from ICU trials, perioperative randomized trials, and physiologic studies consistently demonstrate increased peri-intubation hypoxemia risk and support recruitment-focused preoxygenation. Observational ICU studies report higher rates of severe desaturation and first-pass failure in obese patients, while physiologic studies show rapid oxygen depletion during apnea, despite adequate preoxygenation saturation. Randomized trials in critically ill adults and perioperative obese populations demonstrate that positive airway pressure strategies and head-elevated positioning improve oxygenation and prolong safe apnea time compared with facemask oxygen alone, supporting their use in this phenotype [29,30,31,32].

#### 6.1.4. Recommended Strategy

Recruitment-focused preoxygenation should be the preferred first-line approach in obese patients when feasible. Noninvasive ventilation with PEEP, typically 5 to 10 cm H_2_O, increases functional residual capacity, counteracts atelectasis, and improves ventilation-perfusion matching prior to induction. Head-elevated or ramped positioning should be routinely employed to further augment lung volume and improve airway mechanics. High-flow nasal cannula may be used as an adjunct to maintain apneic oxygenation during laryngoscopy or as an alternative when NIV is poorly tolerated or contraindicated, but it should not replace positive-pressure strategies when recruitment is required. Controlled bag-mask ventilation during induction should be strongly considered to prevent early derecruitment in patients with marginal reserve. Continuous monitoring of oxygen saturation and capnography is critical in patients with obesity [12,33].

### 6.2. Neuromuscular Disease

#### 6.2.1. Why Preoxygenation Fails

Patients with neuromuscular disease are at high risk for peri-intubation hypoxemia because of reduced inspiratory muscle strength and diaphragmatic excursion limit baseline lung volume and ventilatory reserve. Ineffective cough and secretion retention further impair gas exchange and airway patency, such that many patients present at the time of intubation already near their limits of compensation. Loss of residual muscle tone following sedation or neuromuscular blockade precipitates abrupt derecruitment and eliminates the ability to augment tidal volume or respiratory rate. As a result, oxygen stores are rapidly depleted once apnea begins, and desaturation may occur within seconds, despite apparently adequate preoxygenation. Hypercapnia is common and may further destabilize cardiopulmonary physiology during induction [34,35,36].

#### 6.2.2. Dominant Physiologic Constraints

- Reduced baseline FRC and inspiratory capacity;
- Rapid derecruitment after loss of muscle tone;
- Inability to compensate with increased tidal volume or respiratory rate;
- Impaired airway clearance and secretion burden [34,35,36].

Together, these constraints make passive oxygen delivery insufficient and favor strategies that preserve lung volume and minimize apnea throughout the peri-induction period.

#### 6.2.3. Evidence and Applicability

There are no randomized trials specifically evaluating preoxygenation strategies for emergent intubation in patients with neuromuscular disease. Recommendations are therefore extrapolated from ICU and emergency department trials of preoxygenation modalities and from the neuromuscular respiratory failure literature. Across randomized trials in critically ill adults, strategies incorporating positive airway pressure or high-flow oxygen reduce severe peri-intubation hypoxemia compared with conventional facemask oxygen. Although neuromuscular-specific subgroup analyses are lacking, these findings are particularly applicable, given the shared features of markedly reduced respiratory reserve, rapid derecruitment after induction, and poor tolerance of even brief apnea [37].

#### 6.2.4. Recommended Strategy

Patients with neuromuscular weakness should be considered very high risk for peri-intubation hypoxemia and hypercapnia. Preoxygenation strategies that preserve lung volume and support ventilation, such as noninvasive ventilation with PEEP and pressure support, are physiologically preferred when tolerated. Head-elevated or ramped positioning should be routinely employed to optimize diaphragmatic mechanics and reduce early atelectasis. High-flow nasal cannula may serve as an adjunct or alternative when mask-based ventilation is poorly tolerated or when aspiration risk is increased, with the recognition that oxygen delivery alone does not correct ventilatory insufficiency. Controlled bag-mask ventilation during induction may further limit derecruitment and hypercapnia in selected patients with marginal reserve [37].

Given the high likelihood of post-intubation ventilatory dependence and extubation failure in neuromuscular disease, preoxygenation planning should be integrated with the post-intubation respiratory support strategy rather than treated as an isolated peri-procedural step. In these patients, monitoring with capnography is important to identify early respiratory failure before it is evident in oxygen saturation. Continuous capnography can provide early indications of ventilatory failure prior to oxygen desaturation in patients with neuromuscular weakness. A rapid rise in end-tidal CO_2_ or progressive attenuation of the waveform may indicate worsening hypoventilation and should prompt immediate reassessment.

### 6.3. Right Ventricular Failure and Pulmonary Hypertension

#### 6.3.1. Why Preoxygenation Fails

Patients with right ventricular failure and/or pulmonary hypertension represent one of the most physiologically fragile populations undergoing emergent intubation. In this phenotype, peri-intubation hypoxemia is dangerous not only because of limited reserve, but because even brief hypoxemia, hypercapnia, or acidosis can acutely increase pulmonary vascular resistance and overwhelm right ventricular contractile reserve. At baseline, the right ventricle operates near its functional limit, pumping against elevated afterload with minimal capacity to augment output. During apnea, progressive hypoxemia and rising PaCO_2_ increase pulmonary vascular resistance, while sedation and the transition to positive-pressure ventilation reduce venous return and systemic arterial pressure. These combined effects can reduce right ventricular coronary perfusion, precipitating acute dilation, interventricular septal shift, and abrupt loss of left ventricular preload [11].

As a result, safe apnea time is markedly shortened in this population. Clinical deterioration may occur before profound oxygen desaturation is observed, as hypotension and right ventricular failure can precede or outpace changes in pulse oximetry. Even brief apneic intervals that would be tolerated in other critically ill patients may trigger rapid cardiovascular collapse in those with significant pulmonary hypertension or right ventricular dysfunction [38].

#### 6.3.2. Dominant Physiologic Constraints

- Extreme sensitivity to hypoxemia, hypercapnia, and acidosis with rapid PVR rise;
- Sedation and positive-pressure-related reductions in preload and MAP;
- Limited RV contractile reserve and impaired coronary perfusion [11].

Together, these constraints favor airway strategies that minimize apnea, preserve preload and systemic pressure, and avoid abrupt changes in RV afterload.

#### 6.3.3. Evidence and Applicability

There are no randomized trials specifically evaluating preoxygenation strategies in patients with right ventricular failure or pulmonary hypertension undergoing emergent intubation. Available guidance is therefore extrapolated from randomized trials of preoxygenation modalities, observational studies in pulmonary hypertension populations, and the perioperative and critical care literature addressing airway management in right ventricular failure. Observational data consistently demonstrate higher rates of peri-intubation cardiovascular instability and worse outcomes in patients with pulmonary hypertension, underscoring the narrow physiologic margin during apnea. Although right ventricular-specific subgroup analyses are lacking, randomized trials of critically ill adults show that advanced preoxygenation strategies reduce severe peri-intubation hypoxemia compared with conventional oxygen, a finding that is highly relevant in patients in whom hypoxemia and derecruitment can rapidly destabilize right ventricular function [4,10,11].

#### 6.3.4. Recommended Strategy

Patients with significant right ventricular failure or pulmonary hypertension should be considered at extreme risk for peri-intubation cardiopulmonary collapse. Whenever feasible, airway strategies that minimize or eliminate apnea, such as awake or minimally sedated intubation, should be strongly considered to preserve spontaneous ventilation and venous return. When apnea cannot be avoided, preoxygenation should be optimized using modalities that maximize oxygen stores while limiting abrupt changes in preload and afterload. Noninvasive ventilation or high-flow nasal cannula is generally preferred over simple facemask oxygen, with careful attention to patient tolerance and hemodynamic response. Noninvasive ventilation may improve oxygenation but should be applied cautiously, using the lowest effective pressures to avoid excessive intrathoracic pressure and further increases in pulmonary vascular resistance [11].

High-flow nasal cannula provides continuous oxygen delivery and apneic oxygenation during laryngoscopy and may be better-tolerated in unstable or agitated patients [46]. Head-elevated positioning should be maintained throughout preoxygenation and laryngoscopy to improve lung mechanics and delay desaturation. Preoxygenation in this phenotype must be paired with aggressive hemodynamic preparation [39]. Vasopressors should be initiated or escalated prior to induction to maintain systemic pressure and right ventricular coronary perfusion. Induction agents and dosing should be selected to minimize hypotension, and hypoxemia, hypercapnia, and acidosis should be actively avoided before, during, and immediately after intubation [11].

In right ventricular failure and pulmonary hypertension, successful preoxygenation is defined not solely by oxygen saturation but by the ability to traverse induction without precipitating acute right ventricular decompensation. Continuous blood pressure monitoring and watching for pulse pressure changes are critical in identifying imminent cardiovascular collapse.

### 6.4. Post-Operative Patients (ENT, Neurosurgical, Thoracic)

#### 6.4.1. Why Preoxygenation Fails

Post-operative deterioration is a common trigger for emergent re-intubation and is frequently driven by reduced lung volume, rapid derecruitment, and superimposed shunt physiology. Across surgical populations, anesthesia-related atelectasis, supine positioning, pain-limited tidal volumes, residual neuromuscular blockade, and impaired cough contribute to markedly reduced functional residual capacity and unstable end-expiratory lung volume. Aspiration, pulmonary edema, pneumonia, or lobar collapse may further increase the shunt fraction, such that oxygen enrichment alone fails to meaningfully increase arterial oxygen content [40].

In addition to impaired gas exchange, post-operative patients often have procedure-specific anatomic or physiologic constraints that limit the feasibility or safety of standard preoxygenation strategies. Upper airway edema, bleeding, distorted anatomy, or fresh surgical reconstructions may impair mask seal or make interface pressure hazardous. In neurosurgical patients, even brief hypoxemia, hypotension, or hypercapnia can worsen secondary brain injury, while after thoracic surgery, severe atelectasis and ventilation–perfusion mismatch may coexist with vulnerability to excessive positive pressure [41].

#### 6.4.2. Dominant Physiologic Constraints

- Reduced FRC and anesthesia-related atelectasis;
- Rapid derecruitment during induction and apnea;
- Frequent shunt physiology from aspiration, edema, pneumonia, and lung collapse;
- Procedure-specific anatomic or interface constraints [40,41].

Together, these constraints favor recruitment-focused strategies when safe and uninterrupted oxygen delivery through the peri-intubation window.

#### 6.4.3. Evidence and Applicability

Direct randomized evidence focused exclusively on emergent post-operative re-intubation is limited. However, mixed ICU and emergency department trials and meta-analyses in acute hypoxemic respiratory failure, a category that includes many post-operative decompensations, consistently demonstrate that advanced preoxygenation strategies outperform conventional facemask or non-rebreather oxygen [4]. Across these studies, noninvasive ventilation generally provides greater pre-induction oxygenation and reduces the depth of peri-intubation desaturation when compared with conventional oxygen, while high-flow nasal cannula reduces hypoxemic events and enables uninterrupted apneic oxygenation during laryngoscopy [19]. Although surgical subgroups are not consistently reported, these findings are highly applicable given the predominance of atelectasis and shunt physiology in post-operative respiratory failure [40].

#### 6.4.4. Recommended Strategy

Post-operative patients should be treated as high risk for rapid desaturation, with strategy selection guided by the dominant physiology and procedure-specific constraints [40]. Head-elevated positioning should be used whenever feasible [39]. Advanced preoxygenation should be favored over conventional facemask oxygen alone, using noninvasive ventilation when recruitment is required and interface application is safe, and using high-flow nasal cannula when noninvasive ventilation is contraindicated, poorly tolerated, or interface pressure is undesirable. Continuous oxygen delivery during laryngoscopy should be maintained to support apneic oxygenation, and controlled bag-mask ventilation during induction may be appropriate in selected patients with marginal reserve when aspiration risk is acceptable [4,19].

Procedure-specific constraints further refine strategy choice. In ENT post-operative patients, unstable upper airway anatomy, edema, bleeding, or recent reconstruction, HFNC is often favored to avoid compressive mask pressure while maintaining high inspired oxygen concentration and apneic oxygenation [42]. In neurosurgical patients, preoxygenation must avoid the secondary injury triad of hypoxemia, hypotension, and hypercapnia, with strategy selection balancing recruitment needs against tolerance and secretion burden [43]. After thoracic surgery, patients should be presumed atelectasis and shunt dominant, favoring recruitment-based strategies for severe hypoxemia while using high-flow nasal cannula when tolerance, aspiration risk, or concern about excessive positive pressure predominates [44].

In post-operative decompensation, optimal preoxygenation is determined by the interaction between shunt and derecruitment physiology and procedure-specific constraints, reinforcing the value of a phenotype-based approach. Monitoring both oxygen saturation and hemodynamic parameters is essential in maintaining stability for these patients in the peri-intubation period.

## 7. Practical Pitfalls, Tradeoffs, and Failure Modes in Preoxygenation

Although preoxygenation is a cornerstone of safe, emergent intubation, it is frequently ineffective or incomplete in critically ill patients. Failure to recognize and mitigate common pitfalls can result in persistent hypoxemia, hemodynamic collapse, or aspiration, despite apparent adherence to recommended strategies. Understanding these failure modes is essential for translating physiologic principles into reliable bedside practice [45].

### 7.1. Inadequate or Ineffective Preoxygenation

A common and preventable cause of peri-intubation hypoxemia is proceeding to induction without achieving meaningful preoxygenation, a failure that is often operational rather than physiologic in critically ill patients. This typically reflects inadequate time, inadequate delivery, or lack of response monitoring [45].

Inadequate delivery most often originates from ineffective interface application and an inability to sustain a high inspired oxygen concentration during spontaneous breathing. Abbreviated preoxygenation time due to perceived urgency is also common and may occur despite clinical signs that oxygenation is not improving. In addition, clinicians may overinterpret transient improvement in pulse oximetry as evidence of adequate oxygen reserve, even when true alveolar denitrogenation and lung volume stabilization have not been achieved [47].

Accordingly, preoxygenation should be treated as an observable intervention with an assessable response. Clinicians should monitor the trajectory and stability of oxygen saturation during preoxygenation, confirm that the chosen interface is achieving the intended physiologic effect, and escalate early when the response is inadequate. When preoxygenation remains suboptimal, delaying induction briefly to correct delivery problems or transition to a more effective strategy is often safer than proceeding into apnea with an unacceptably narrow margin.

### 7.2. Patient Factors That Limit Preoxygenation Benefit

Even when preoxygenation is technically adequate, certain patient-level constraints can leave little physiologic reserve and reduce the benefit of any single modality. These constraints should prompt earlier escalation to multimodal approaches and a plan to minimize apnea time.

#### 7.2.1. Reduced Lung Volume and Rapid Derecruitment

First, severely reduced lung volume and rapid derecruitment, as seen in obesity, neuromuscular weakness, ascites, pregnancy, or strict supine positioning, can cause oxygen stores to deplete quickly once spontaneous ventilation ceases. In these patients, the problem is not only oxygen concentration but also instability of end-expiratory lung volume during the induction window.

#### 7.2.2. Shunt Physiology

Second, severe shunt physiology, such as ARDS, pneumonia, aspiration, or pulmonary edema, limits the ability of oxygen enrichment alone to increase arterial oxygen content. In these states, recruitment and shunt reduction are often more important than further increases in FiO_2_ [40].

#### 7.2.3. Increased Oxygen Consumption

Finally, elevated oxygen consumption from fever, sepsis, agitation, or metabolic acidosis accelerates depletion of oxygen stores during apnea even when preoxygenation is optimized. When these constraints are present, success relies on anticipating rapid failure, minimizing oxygen interruption, and pairing oxygenation strategy selection with a deliberate plan for apnea-limiting tactics and hemodynamic preparation [47].

#### 7.2.4. Anticipated Failure/Troubleshooting

Because these constraints are common and often under-recognized, clinicians should anticipate device-specific failure modes and use structured troubleshooting to preserve oxygenation through the induction window.

### 7.3. Modality-Specific Pitfalls

#### 7.3.1. Reasons for Failure

Preoxygenation failures frequently reflect predictable interface, technique, and sequencing problems rather than an unavoidable physiologic limit. Each modality has characteristic failure modes that can be mitigated with structured troubleshooting.

#### 7.3.2. Conventional Facemask

Conventional facemask oxygen is highly sensitive to leak and room air entrainment and does not stabilize lung volume. Its most common failure is inadequate effective FiO_2_ due to a poor seal, mouth breathing, or high inspiratory demand. When the response is suboptimal, the appropriate correction is not simply higher flow, but escalation to a modality that adds positive pressure, improves seal, or both [27,47].

#### 7.3.3. Bag-Mask Ventilation

Bag-mask ventilation can prevent early oxygen depletion after induction, but failure most often reflects airway obstruction, poor mask seal, or excessive ventilation. Use of an oral airway, two-person technique, and gentle pressures with attention to chest rise improves effectiveness while limiting gastric insufflation and hemodynamic compromise [14].

#### 7.3.4. Noninvasive Ventilation

Noninvasive ventilation provides recruitment and shunt reduction, but failures usually arise from intolerance, asynchronous breathing, or hemodynamic sensitivity to pressure. In high-risk patients, the key pitfalls are excessive pressures and abrupt discontinuation at induction without a plan to maintain lung volume or apneic oxygenation. When NIV must be removed, maintaining HFNC or nasal cannula oxygen during laryngoscopy and limiting apnea time can reduce rapid post-induction desaturation [19].

#### 7.3.5. High-Flow Nasal Canula

HFNC is best understood as a continuity tool rather than a recruitment strategy. Its main failure mode is over-reliance in patients with severe shunt or derecruitment-predominant physiology, where oxygen delivery without lung volume stabilization can provide a false sense of security. In these patients, HFNC should be used as an adjunct for apneic oxygenation rather than as a standalone preoxygenation strategy [46].

### 7.4. Hemodynamic Consequences of the Peri-Induction Transition

Peri-intubation hemodynamic instability is common, occurring in approximately 40–45% of emergent ICU and ED intubations, and is strongly associated with mortality. While preoxygenation strategies primarily target hypoxemia, their interaction with cardiopulmonary mechanics can meaningfully influence hemodynamic risk [45].

Transition from spontaneous breathing to positive-pressure ventilation reduces venous return and may precipitate hypotension, particularly in hypovolemic or vasoplegic states. Aggressive recruitment or hyperventilation during preoxygenation increases intrathoracic pressure and can worsen shock or precipitate right ventricular failure. Hypoxemia itself is a major trigger for peri-intubation cardiac arrest, linking ineffective preoxygenation directly to hemodynamic collapse [38].

The preoxygenation window, therefore, represents a critical opportunity for parallel hemodynamic optimization. Early initiation or escalation of vasopressors, judicious fluid administration when appropriate, and avoidance of excessive induction agent dosing are important adjuncts to oxygenation strategy selection [45].

### 7.5. Aspiration Risk and Airway-Related Tradeoffs

Aspiration remains a concern during emergent intubation, particularly in non-fasted patients or those with impaired airway reflexes. However, recent ICU trials demonstrate that, when performed with a careful technique, positive-pressure strategies such as BMV and NIV do not measurably increase clinically evident aspiration compared with oxygen mask preoxygenation or no ventilation. Reported aspiration rates across randomized ICU studies are low, generally 1–4%, and are not higher with positive-pressure approaches [14].

Aspiration risk increases with high inspiratory pressures, large tidal volumes, poor mask fit, prolonged positive-pressure ventilation, and supine positioning. In contrast, gentle ventilation with low-to-moderate pressures, head-elevated positioning, and suction readiness appears to keep the aspiration risk low while substantially reducing the far more frequent and dangerous complications of hypoxemia and cardiovascular collapse. In practical terms, the balance of evidence favors prioritizing the prevention of severe hypoxemia in high-risk patients while mitigating the aspiration risk through technique and positioning [14,41].

### 7.6. Hyperoxemia: Contextual Risk and Practical Management

Sustained hyperoxemia has been associated with adverse outcomes in mechanically ventilated and critically ill populations, including lung injury, vasoconstriction, and worse neurologic outcomes in selected groups. However, during emergent intubation, the immediate threat posed by severe hypoxemia far outweighs the theoretical risk of brief exposure to high inspired oxygen concentrations [48]. Accordingly, the use of FiO_2_ 1.0 during preoxygenation and the apneic phase is appropriate in high-risk patients. The principal hyperoxia-related harm arises from failure to de-escalate oxygen therapy after airway control is achieved. Prompt titration of FiO_2_ to physiologic targets once the patient is stabilized, particularly in post–cardiac arrest, severe traumatic brain injury, or stroke, is essential to minimize sustained hyperoxemia [27].

### 7.7. Process and Human-Factor Failures

Finally, many preoxygenation failures reflect system and cognitive factors rather than physiology alone. Failure to monitor response during preoxygenation, delayed escalation from ineffective strategies, proceeding to paralysis despite inadequate oxygenation, and a lack of parallel hemodynamic preparation are common and preventable errors. Interface intolerance, mask leak, and agitation account for a substantial proportion of failed preoxygenation in ICU trials, with protocol-defined failure rates of assigned strategies on the order of 10–20% [19,45].

Structured preparation, early troubleshooting, and sedation-assisted preoxygenation in selected patients can mitigate these failures and improve the reliability of preoxygenation in real-world practice [19,45].

## 8. Future Directions

Significant knowledge gaps persist regarding the optimal peri-intubation management of critically ill patients. Future research should assess whether a phenotype-directed approach effectively reduces the incidence of peri-intubation hypoxemia and cardiovascular collapse. Prospective trials should prioritize physiological endpoints, specifically oxygen reserve, apnea tolerance, and circulatory stability, within these distinct populations. Furthermore, emerging technologies such as artificial intelligence (AI)-assisted monitoring platforms offer substantial promise. The real-time integration of capnography, hemodynamic variables, and oxygenation via AI may transition airway management from a reactive approach to a predictive model. These tools could enable early recognition of phenotypes predisposed to decompensation, providing clinicians with proactive warnings before instability occurs. Ultimately, research must pivot from the comparison of individual preoxygenation devices toward prioritizing patient-specific risk characteristics and interventions designed to preemptively mitigate the risk of peri-procedural arrest.

## 9. Conclusions

A phenotype-based approach reframes preoxygenation from “which device is available” to “which physiologic constraint is dominant,” enabling the intentional selection of recruitment, continuous oxygen delivery, and controlled ventilation strategies while proactively anticipating failure modes. Applying this framework, particularly in high-risk populations, can extend safe apnea time, mitigate severe desaturation, and support hemodynamic stability during emergent airway management.

## Figures and Tables

**Table 1:** Determinants of rapid peri-intubation hypoxemia.

Physiologic Derangement	Mechanism	Clinical Manifestation	Implication for Preoxygenation
Reduced FRC [5,6,7]	Atelectasis Pulmonary edema Positioning	Rapid collapse of dependent lung units Precipitous desaturation	Decreased apnea time
Intrapulmonary Shunt/ VQ mismatch [3,6]	Loss of perfusion to ventilated alveoli and derecruitment	Hypoxia despite oxygen enrichment	Need for recruitment strategies
Increased oxygen consumption/ Limited metabolic reserve [8,9]	↑ systemic demand secondary to underlying critical condition	Rapid depletion of oxygen stores	Decreased apnea time
Hemodynamic instability [10,11]	↓ Cardiac output ↓ Mixed venous oxygen saturation ↓ Preload ↓ Systemic arterial pressure	↑ RV ischemia Interventricular septal shift Abrupt cardiovascular collapse	Decreased apnea tolerance
Procedural amplifiers [5,6,7]	Sedation Neuromuscular blockade Prolonged laryngoscopy attempts Suboptimal positioning interruptions in oxygen delivery	↑ Derecruitment	Decreased apnea time

↑ = Increased; ↓ = Decreased.

**Table 2:** Summary of key clinical trials informing evidence-based preoxygenation in the critically ill. BMV: bag-mask ventilation; ED: emergency department; HFNC: high-flow nasal Cannula; ICU: intensive care unit; NIV: noninvasive ventilation; NRB: non-rebreather mask; SpO_2_: peripheral oxygen saturation.

Trial	Population	Intervention	Comparison	Outcome	Key Takeaway
Baillard et al. (2006) [15]	Hypoxemic ICU patients requiring intubation	NIV	Standard O_2_ mask	Severe desaturation in peri-intubation period	Positive-pressure ventilation significantly reduces peri-intubation hypoxemia
OPTINIV (2016) [16]	Hypoxemic ICU patients requiring intubation	HFNC + NIV	NIV alone	Lowest SpO_2_	Combining recruitment (NIV) with apneic oxygenation (HFNC) further optimizes nadir SpO_2_
PROTRACH [17]	ICU patients requiring intubation	HFNC	BMV	Lowest SpO_2_	No significant difference between HFNC and BMV in preventing desaturation
PREOYFLOW (2017) [18]	ICU patients requiring intubation	HFNC	NRB mask	Lowest SpO_2_	HFNC alone was not sufficient in patients with high-risk physiology
PREVENT (2019) [14]	ICU patients requiring intubation	BMV	No pre-oxygenation	Lowest SpO_2_	BMV had lower incidence of hypoxemia and a higher lowest SpO_2_
PREOXI (2024) [19]	ICU and ED patients requiring emergent intubation	NIV	NRB or Standard O_2_ mask	Hypoxemia (<85% SpO_2_) during intubation	NIV preoxygenation significantly reduces the risk of hypoxemia during emergent intubation compared to conventional mask oxygen

**Table 3:** Phenotype-based preoxygenation strategies. ALS: amyotrophic lateral sclerosis; FRC: functional residual capacity; HFNC: high-flow nasal cannula; IC: inspiratory capacity; MAP: mean arterial pressure; NIPPV: noninvasive positive-pressure ventilation; O_2_: oxygen; PVR: pulmonary vascular resistance; RR: respiratory rate; RV: right ventricle; VT: tidal volume.

Clinical Context	Patient Phenotype	Dominant Physiologic Constraint	Preferred Preoxygenation Strategy (Multimodal)	Adjuncts	Key Evidence	Key Caveats
Morbidly obese patient with acute pneumonia and significant abdominal distention.	Obesity	↓ FRC ↑ O_2_ consumption Rapid derecruitment Dependent lung physiology	NIPPV for recruitment followed by HFNC for apneic oxygenation.	Ramped positioning	[12,29,30,31,32,33]	Tolerance of NIPPV
Patient with myasthenic crisis or ALS presenting with weak cough and hypercapnia.	Neuromuscular weakness	↓ Baseline FRC/IC Rapid derecruitment Inability to compensate (↑ VT/RR) Impaired airway clearance/↑ secretion burden	NIPPV for volume stabilization paired with controlled bag-mask ventilation during the induction interval.	Ramped positioning HFNC during laryngoscopy	[34,35,36,37]	Mask-based ventilation alone doesn’t correct for hypercapnia
Decompensated pulmonary hypertension patient with septic shock and RV dilation.	Pulmonary Hypertension/ RV failure [4,10,11,38]	Sensitivity to hypoxemia, hypercapnia, and acidosis with rapid PVR rise. Sedation and positive-pressure-related reductions in preload and MAP. Limited RV contractile reserve and impaired coronary perfusion.	Minimal sedation HFNC or cautious NIPPV to preserve spontaneous ventilation and preload.	Ramped positioning Aggressive hemodynamic monitoring and rapid initiation/escalation of pressors	[4,10,11,38]	Reduction in peri-intubation hypoxemia with advanced preoxygenation techniques helps prevent rapid destabilization of RV function
Post-thoracotomy patient with lobar collapse, anesthesia-related atelectasis, and hypoxia.	Post-operative respiratory failure	↓ FRC Rapid derecruitment ↑ Shunt physiology Procedure-specific constraints	Procedure-dependent; NIPPV for thoracic shunt followed by HFNC for continuous delivery during laryngoscopy.	Procedure-specific constraints vs. shunt physiology of derecruitment	[4,19,39,40,41,42,43,44,45]	Decisions should be procedure and patient-specific

↑ = Increased; ↓ = Decreased.

## Data Availability

All data can be found through a literature search utilizing the methods detailed in the Section 2 of this manuscript.
